# Supplementary material for: Heparin-based hydrogel scaffolding alters the transcriptomic profile and increases the chemoresistance of MDA-MB-231 triple-negative breast cancer cells
Source: Biomater Sci. 2020 Feb 13;8(10):2786–96. doi: 10.1039/c9bm01481k (PMC7497406; doi:10.1039/c9bm01481k)
Supplement: Supplementary file 2 [file BM-008-C9BM01481K-s002.zip › Supplementary File 4/HP-BhydrogelvControl/Pathways/my_analysis.Gsea.1545201079907/HALLMARK_OXIDATIVE_PHOSPHORYLATION.html]

Details for gene set HALLMARK\_OXIDATIVE\_PHOSPHORYLATION[GSEA]

|  || Dataset | expr.class.cls#HYD\_versus\_CONTROL.class.cls#HYD\_versus\_CONTROL\_repos |
| Phenotype | class.cls#HYD\_versus\_CONTROL\_repos |
| Upregulated in class | CONTROL |
| GeneSet | HALLMARK\_OXIDATIVE\_PHOSPHORYLATION |
| Enrichment Score (ES) | -0.6635401 |
| Normalized Enrichment Score (NES) | -3.1634915 |
| Nominal p-value | 0.0 |
| FDR q-value | 0.0 |
| FWER p-Value | 0.0 |
Table: GSEA Results Summary

  

Fig 1: Enrichment plot: HALLMARK\_OXIDATIVE\_PHOSPHORYLATION      
 Profile of the Running ES Score & Positions of GeneSet Members on the Rank Ordered List

  

| PROBE | DESCRIPTION (from dataset) | GENE SYMBOL | GENE\_TITLE | RANK IN GENE LIST | RANK METRIC SCORE | RUNNING ES | CORE ENRICHMENT || 1 | RHOT2 | na |  |  | 1503 | 1.574 | -0.0715 | No |
| 2 | ACADVL | na |  |  | 1828 | 1.430 | -0.0817 | No |
| 3 | TCIRG1 | na |  |  | 1902 | 1.406 | -0.0788 | No |
| 4 | PMPCA | na |  |  | 2012 | 1.356 | -0.0780 | No |
| 5 | NDUFV2 | na |  |  | 2147 | 1.296 | -0.0788 | No |
| 6 | ACAA1 | na |  |  | 3257 | 0.975 | -0.1325 | No |
| 7 | ATP6V0C | na |  |  | 3320 | 0.961 | -0.1312 | No |
| 8 | CPT1A | na |  |  | 3541 | 0.908 | -0.1384 | No |
| 9 | ECI1 | na |  |  | 4503 | 0.716 | -0.1855 | No |
| 10 | SURF1 | na |  |  | 4879 | 0.645 | -0.2021 | No |
| 11 | NDUFS7 | na |  |  | 4921 | 0.638 | -0.2012 | No |
| 12 | BAX | na |  |  | 4924 | 0.638 | -0.1983 | No |
| 13 | MFN2 | na |  |  | 5171 | 0.604 | -0.2083 | No |
| 14 | NDUFA9 | na |  |  | 5342 | 0.579 | -0.2145 | No |
| 15 | POR | na |  |  | 5382 | 0.572 | -0.2138 | No |
| 16 | MTRR | na |  |  | 5398 | 0.570 | -0.2118 | No |
| 17 | NDUFV1 | na |  |  | 5555 | 0.544 | -0.2174 | No |
| 18 | NDUFB2 | na |  |  | 6422 | 0.427 | -0.2610 | No |
| 19 | IDH3G | na |  |  | 7740 | 0.282 | -0.3289 | No |
| 20 | FDX1 | na |  |  | 7920 | 0.259 | -0.3371 | No |
| 21 | ETFB | na |  |  | 8173 | 0.236 | -0.3492 | No |
| 22 | TIMM50 | na |  |  | 8666 | 0.184 | -0.3742 | No |
| 23 | AFG3L2 | na |  |  | 8677 | 0.182 | -0.3738 | No |
| 24 | CYC1 | na |  |  | 8715 | 0.179 | -0.3749 | No |
| 25 | RHOT1 | na |  |  | 9058 | 0.146 | -0.3922 | No |
| 26 | AIFM1 | na |  |  | 9365 | 0.109 | -0.4078 | No |
| 27 | ECH1 | na |  |  | 9762 | 0.069 | -0.4283 | No |
| 28 | SLC25A6 | na |  |  | 9796 | 0.065 | -0.4297 | No |
| 29 | TIMM8B | na |  |  | 10120 | 0.026 | -0.4466 | No |
| 30 | PDHX | na |  |  | 10183 | 0.017 | -0.4498 | No |
| 31 | SDHA | na |  |  | 10187 | 0.016 | -0.4499 | No |
| 32 | RETSAT | na |  |  | 10420 | 0.005 | -0.4620 | No |
| 33 | ATP5D | na |  |  | 10670 | -0.026 | -0.4750 | No |
| 34 | HSD17B10 | na |  |  | 10723 | -0.032 | -0.4776 | No |
| 35 | TIMM13 | na |  |  | 11028 | -0.070 | -0.4933 | No |
| 36 | GPI | na |  |  | 11431 | -0.105 | -0.5139 | No |
| 37 | SLC25A11 | na |  |  | 11717 | -0.140 | -0.5282 | No |
| 38 | TIMM9 | na |  |  | 12297 | -0.213 | -0.5577 | No |
| 39 | ACO2 | na |  |  | 12768 | -0.255 | -0.5812 | No |
| 40 | GOT2 | na |  |  | 12892 | -0.271 | -0.5864 | No |
| 41 | COX10 | na |  |  | 12934 | -0.275 | -0.5872 | No |
| 42 | MRPS15 | na |  |  | 13061 | -0.292 | -0.5924 | No |
| 43 | COX8A | na |  |  | 13216 | -0.312 | -0.5990 | No |
| 44 | MAOB | na |  |  | 13304 | -0.326 | -0.6020 | No |
| 45 | LDHB | na |  |  | 13307 | -0.326 | -0.6006 | No |
| 46 | PDP1 | na |  |  | 13308 | -0.327 | -0.5990 | No |
| 47 | SLC25A20 | na |  |  | 13468 | -0.348 | -0.6057 | No |
| 48 | DLST | na |  |  | 13510 | -0.354 | -0.6062 | No |
| 49 | ATP1B1 | na |  |  | 13610 | -0.371 | -0.6096 | No |
| 50 | COX11 | na |  |  | 13687 | -0.384 | -0.6117 | No |
| 51 | CYB5R3 | na |  |  | 13709 | -0.387 | -0.6110 | No |
| 52 | ATP6AP1 | na |  |  | 13746 | -0.395 | -0.6110 | No |
| 53 | TOMM22 | na |  |  | 13809 | -0.406 | -0.6123 | No |
| 54 | NNT | na |  |  | 14077 | -0.428 | -0.6243 | No |
| 55 | GRPEL1 | na |  |  | 14110 | -0.430 | -0.6239 | No |
| 56 | FXN | na |  |  | 14201 | -0.441 | -0.6265 | No |
| 57 | MRPL11 | na |  |  | 14251 | -0.453 | -0.6269 | No |
| 58 | VDAC3 | na |  |  | 14267 | -0.455 | -0.6255 | No |
| 59 | IDH2 | na |  |  | 14279 | -0.459 | -0.6239 | No |
| 60 | NDUFS8 | na |  |  | 14282 | -0.459 | -0.6218 | No |
| 61 | GPX4 | na |  |  | 14284 | -0.460 | -0.6197 | No |
| 62 | NQO2 | na |  |  | 14292 | -0.462 | -0.6178 | No |
| 63 | OGDH | na |  |  | 14347 | -0.470 | -0.6184 | No |
| 64 | COX15 | na |  |  | 14357 | -0.471 | -0.6166 | No |
| 65 | NDUFA5 | na |  |  | 14435 | -0.481 | -0.6183 | No |
| 66 | CS | na |  |  | 14588 | -0.504 | -0.6239 | No |
| 67 | SLC25A3 | na |  |  | 14602 | -0.506 | -0.6222 | No |
| 68 | ATP5F1 | na |  |  | 14725 | -0.527 | -0.6261 | No |
| 69 | IDH3B | na |  |  | 14744 | -0.530 | -0.6245 | No |
| 70 | MTRF1 | na |  |  | 14841 | -0.543 | -0.6269 | No |
| 71 | NDUFS3 | na |  |  | 14957 | -0.555 | -0.6303 | No |
| 72 | NDUFC2 | na |  |  | 14994 | -0.561 | -0.6295 | No |
| 73 | LRPPRC | na |  |  | 15236 | -0.603 | -0.6393 | No |
| 74 | PDK4 | na |  |  | 15248 | -0.606 | -0.6369 | No |
| 75 | PDHA1 | na |  |  | 15305 | -0.618 | -0.6369 | No |
| 76 | OXA1L | na |  |  | 15411 | -0.636 | -0.6394 | No |
| 77 | ATP5G3 | na |  |  | 15609 | -0.670 | -0.6465 | No |
| 78 | PHB2 | na |  |  | 15698 | -0.681 | -0.6479 | No |
| 79 | UQCR11 | na |  |  | 15707 | -0.683 | -0.6450 | No |
| 80 | SLC25A12 | na |  |  | 15836 | -0.711 | -0.6484 | No |
| 81 | NDUFA3 | na |  |  | 16123 | -0.773 | -0.6597 | No |
| 82 | ECHS1 | na |  |  | 16197 | -0.787 | -0.6598 | Yes |
| 83 | ETFA | na |  |  | 16240 | -0.797 | -0.6581 | Yes |
| 84 | ISCU | na |  |  | 16260 | -0.800 | -0.6553 | Yes |
| 85 | MRPS22 | na |  |  | 16285 | -0.807 | -0.6527 | Yes |
| 86 | SLC25A4 | na |  |  | 16335 | -0.818 | -0.6513 | Yes |
| 87 | ACAT1 | na |  |  | 16339 | -0.818 | -0.6476 | Yes |
| 88 | ALAS1 | na |  |  | 16364 | -0.825 | -0.6449 | Yes |
| 89 | MRPL35 | na |  |  | 16409 | -0.834 | -0.6432 | Yes |
| 90 | DLD | na |  |  | 16423 | -0.837 | -0.6398 | Yes |
| 91 | ACADM | na |  |  | 16495 | -0.853 | -0.6395 | Yes |
| 92 | DECR1 | na |  |  | 16501 | -0.855 | -0.6356 | Yes |
| 93 | DLAT | na |  |  | 16528 | -0.860 | -0.6329 | Yes |
| 94 | COX7C | na |  |  | 16533 | -0.862 | -0.6290 | Yes |
| 95 | CYCS | na |  |  | 16645 | -0.885 | -0.6305 | Yes |
| 96 | NDUFB3 | na |  |  | 16671 | -0.891 | -0.6276 | Yes |
| 97 | TIMM10 | na |  |  | 16690 | -0.896 | -0.6242 | Yes |
| 98 | CYB5A | na |  |  | 16738 | -0.910 | -0.6223 | Yes |
| 99 | NDUFS1 | na |  |  | 16749 | -0.913 | -0.6185 | Yes |
| 100 | COX7A2L | na |  |  | 16777 | -0.921 | -0.6155 | Yes |
| 101 | HTRA2 | na |  |  | 16878 | -0.948 | -0.6162 | Yes |
| 102 | BDH2 | na |  |  | 16884 | -0.950 | -0.6119 | Yes |
| 103 | SLC25A5 | na |  |  | 16915 | -0.961 | -0.6088 | Yes |
| 104 | MDH1 | na |  |  | 16955 | -0.971 | -0.6062 | Yes |
| 105 | MRPS11 | na |  |  | 16973 | -0.978 | -0.6024 | Yes |
| 106 | NDUFA6 | na |  |  | 16977 | -0.979 | -0.5979 | Yes |
| 107 | TIMM17A | na |  |  | 17041 | -0.997 | -0.5964 | Yes |
| 108 | UQCRC1 | na |  |  | 17067 | -1.006 | -0.5929 | Yes |
| 109 | OPA1 | na |  |  | 17077 | -1.009 | -0.5885 | Yes |
| 110 | HADHA | na |  |  | 17085 | -1.011 | -0.5840 | Yes |
| 111 | IDH3A | na |  |  | 17098 | -1.014 | -0.5798 | Yes |
| 112 | NDUFS6 | na |  |  | 17118 | -1.020 | -0.5759 | Yes |
| 113 | HADHB | na |  |  | 17247 | -1.066 | -0.5775 | Yes |
| 114 | ATP5G1 | na |  |  | 17278 | -1.076 | -0.5739 | Yes |
| 115 | UQCRB | na |  |  | 17279 | -1.077 | -0.5687 | Yes |
| 116 | PHYH | na |  |  | 17292 | -1.081 | -0.5642 | Yes |
| 117 | ATP6V0E1 | na |  |  | 17388 | -1.117 | -0.5638 | Yes |
| 118 | NDUFB7 | na |  |  | 17428 | -1.125 | -0.5605 | Yes |
| 119 | HSPA9 | na |  |  | 17460 | -1.137 | -0.5566 | Yes |
| 120 | ATP6V0B | na |  |  | 17496 | -1.152 | -0.5529 | Yes |
| 121 | SUPV3L1 | na |  |  | 17567 | -1.183 | -0.5509 | Yes |
| 122 | ATP6V1F | na |  |  | 17588 | -1.191 | -0.5463 | Yes |
| 123 | MDH2 | na |  |  | 17620 | -1.207 | -0.5421 | Yes |
| 124 | ATP6V1D | na |  |  | 17625 | -1.209 | -0.5365 | Yes |
| 125 | IMMT | na |  |  | 17677 | -1.232 | -0.5333 | Yes |
| 126 | ATP5H | na |  |  | 17694 | -1.243 | -0.5282 | Yes |
| 127 | ATP5A1 | na |  |  | 17706 | -1.244 | -0.5228 | Yes |
| 128 | NDUFAB1 | na |  |  | 17728 | -1.254 | -0.5178 | Yes |
| 129 | NDUFB5 | na |  |  | 17730 | -1.255 | -0.5119 | Yes |
| 130 | HCCS | na |  |  | 17784 | -1.280 | -0.5085 | Yes |
| 131 | VDAC2 | na |  |  | 17787 | -1.281 | -0.5025 | Yes |
| 132 | NDUFS2 | na |  |  | 17809 | -1.291 | -0.4974 | Yes |
| 133 | COX7A2 | na |  |  | 17829 | -1.299 | -0.4921 | Yes |
| 134 | ATP5L | na |  |  | 17842 | -1.304 | -0.4865 | Yes |
| 135 | COX4I1 | na |  |  | 17868 | -1.318 | -0.4815 | Yes |
| 136 | SDHC | na |  |  | 17944 | -1.344 | -0.4790 | Yes |
| 137 | ETFDH | na |  |  | 17960 | -1.356 | -0.4733 | Yes |
| 138 | ATP6V1G1 | na |  |  | 17978 | -1.367 | -0.4676 | Yes |
| 139 | UQCRC2 | na |  |  | 17985 | -1.373 | -0.4613 | Yes |
| 140 | ATP6V1H | na |  |  | 18048 | -1.414 | -0.4578 | Yes |
| 141 | IDH1 | na |  |  | 18161 | -1.475 | -0.4566 | Yes |
| 142 | ABCB7 | na |  |  | 18167 | -1.479 | -0.4498 | Yes |
| 143 | PRDX3 | na |  |  | 18171 | -1.480 | -0.4428 | Yes |
| 144 | MGST3 | na |  |  | 18186 | -1.490 | -0.4364 | Yes |
| 145 | MRPL15 | na |  |  | 18190 | -1.490 | -0.4294 | Yes |
| 146 | MRPS12 | na |  |  | 18205 | -1.503 | -0.4229 | Yes |
| 147 | SDHD | na |  |  | 18206 | -1.504 | -0.4157 | Yes |
| 148 | UQCR10 | na |  |  | 18208 | -1.505 | -0.4085 | Yes |
| 149 | CASP7 | na |  |  | 18210 | -1.506 | -0.4013 | Yes |
| 150 | SUCLA2 | na |  |  | 18223 | -1.511 | -0.3947 | Yes |
| 151 | ATP5J | na |  |  | 18225 | -1.514 | -0.3875 | Yes |
| 152 | UQCRQ | na |  |  | 18242 | -1.522 | -0.3810 | Yes |
| 153 | FH | na |  |  | 18267 | -1.540 | -0.3749 | Yes |
| 154 | COX6C | na |  |  | 18290 | -1.558 | -0.3686 | Yes |
| 155 | ATP5B | na |  |  | 18322 | -1.584 | -0.3626 | Yes |
| 156 | ALDH6A1 | na |  |  | 18402 | -1.641 | -0.3589 | Yes |
| 157 | NDUFA2 | na |  |  | 18416 | -1.648 | -0.3516 | Yes |
| 158 | PDHB | na |  |  | 18418 | -1.650 | -0.3438 | Yes |
| 159 | MRPS30 | na |  |  | 18429 | -1.659 | -0.3363 | Yes |
| 160 | COX5A | na |  |  | 18472 | -1.687 | -0.3304 | Yes |
| 161 | MRPL34 | na |  |  | 18541 | -1.736 | -0.3257 | Yes |
| 162 | NDUFC1 | na |  |  | 18543 | -1.739 | -0.3174 | Yes |
| 163 | NDUFA8 | na |  |  | 18544 | -1.740 | -0.3090 | Yes |
| 164 | VDAC1 | na |  |  | 18615 | -1.802 | -0.3040 | Yes |
| 165 | ATP6V1C1 | na |  |  | 18618 | -1.804 | -0.2955 | Yes |
| 166 | COX6B1 | na |  |  | 18634 | -1.825 | -0.2875 | Yes |
| 167 | UQCRFS1 | na |  |  | 18680 | -1.875 | -0.2809 | Yes |
| 168 | UQCRH | na |  |  | 18727 | -1.923 | -0.2741 | Yes |
| 169 | SDHB | na |  |  | 18744 | -1.946 | -0.2655 | Yes |
| 170 | NDUFB6 | na |  |  | 18750 | -1.954 | -0.2564 | Yes |
| 171 | ACADSB | na |  |  | 18752 | -1.955 | -0.2471 | Yes |
| 172 | GLUD1 | na |  |  | 18763 | -1.965 | -0.2382 | Yes |
| 173 | ATP5O | na |  |  | 18790 | -2.007 | -0.2299 | Yes |
| 174 | ACAA2 | na |  |  | 18837 | -2.070 | -0.2224 | Yes |
| 175 | COX17 | na |  |  | 18860 | -2.099 | -0.2135 | Yes |
| 176 | ATP5G2 | na |  |  | 18863 | -2.104 | -0.2035 | Yes |
| 177 | OAT | na |  |  | 18873 | -2.130 | -0.1937 | Yes |
| 178 | NDUFB1 | na |  |  | 18881 | -2.139 | -0.1838 | Yes |
| 179 | ISCA1 | na |  |  | 18884 | -2.139 | -0.1736 | Yes |
| 180 | NDUFB4 | na |  |  | 18890 | -2.151 | -0.1636 | Yes |
| 181 | ATP5C1 | na |  |  | 18950 | -2.263 | -0.1558 | Yes |
| 182 | COX6A1 | na |  |  | 18964 | -2.287 | -0.1455 | Yes |
| 183 | COX5B | na |  |  | 18970 | -2.297 | -0.1347 | Yes |
| 184 | MTX2 | na |  |  | 18971 | -2.307 | -0.1237 | Yes |
| 185 | ATP5E | na |  |  | 19004 | -2.358 | -0.1140 | Yes |
| 186 | ATP6V1E1 | na |  |  | 19023 | -2.415 | -0.1034 | Yes |
| 187 | SUCLG1 | na |  |  | 19027 | -2.431 | -0.0919 | Yes |
| 188 | ATP5I | na |  |  | 19040 | -2.495 | -0.0805 | Yes |
| 189 | NDUFA4 | na |  |  | 19086 | -2.670 | -0.0701 | Yes |
| 190 | NDUFB8 | na |  |  | 19093 | -2.699 | -0.0574 | Yes |
| 191 | NDUFA1 | na |  |  | 19145 | -3.089 | -0.0453 | Yes |
| 192 | NDUFS4 | na |  |  | 19157 | -3.232 | -0.0303 | Yes |
| 193 | ATP5J2 | na |  |  | 19172 | -3.276 | -0.0153 | Yes |
| 194 | COX7B | na |  |  | 19184 | -3.465 | 0.0007 | Yes |
Table: GSEA details [plain text format]

  

Fig 2: HALLMARK\_OXIDATIVE\_PHOSPHORYLATION      
 Blue-Pink O' Gram in the Space of the Analyzed GeneSet

  

Fig 3: HALLMARK\_OXIDATIVE\_PHOSPHORYLATION: Random ES distribution      
 Gene set null distribution of ES for **HALLMARK\_OXIDATIVE\_PHOSPHORYLATION**
